# Dublin's homeless crisis – is this reflected in emergency department psychiatry referrals?

**DOI:** 10.1192/bjo.2021.555

**Published:** 2021-06-18

**Authors:** Aoibheann McLoughlin, Anna Feeney, John Cooney

**Affiliations:** 1 St. James's Hospital; 2 St. Patrick's Hospital

## Abstract

**Aims:**

This study seeks to explore the prevalence and impact of homelessness in an adult sample of psychiatry referrals over a one-month period via the Emergency Department at St. James's Hospital.

**Background:**

Homelessness has now reached a crisis point in Ireland. In July 2019, there were 10,275 people documented as homeless nationwide, with the number of homeless families increasing by 178% since June 2015. The majority of individuals registered as homeless are located in Dublin. St. James's Hospital (SJH) provides psychiatric care to a population of 136,704 people across Dublin South-City within areas of significant deprivation according to the most recent social deprivation index.

**Method:**

All Emergency Department psychiatry referrals over a one-month period were recorded. Month of study was randomly generated. Data were collected from electronic records. Socio-demographic information was analysed. Data were anonymised and recorded using Microsoft Excel. Current homelessness statistics were accessed from the Department of Housing, Planning, and Local Government and compared to the data collected.

**Result:**

During the month of the Study (March 2019), 4315 adults accessed emergency homeless accommodation in Dublin. Of the 109 psychiatry referrals received through the Emergency Department at SJH during this time, over a quarter (28%) of those referred reported themselves to be homeless or living in temporary accommodation. An additional 5% were documented as living in residential or sheltered care at time of assessment. All of the referred homeless patients were unemployed (n = 30). 50% of homeless patients were referred to psychiatry following expressed thoughts or acts of self-harm. Illicit drug abuse was associated with 73% of referrals. Alcohol abuse was associated with 47%. Of those who were referred, under a quarter (23%) were assessed as having a major mental illness, and in the majority of these cases, illicit drug and alcohol abuse were compounding factors in exacerbating symptomatology. Of those referred, 66% had previously been reviewed by psychiatry during prior ED presentations and 60% of homeless presenters reported that they had previously been, or were currently linked in with community mental health teams.

**Conclusion:**

Frequently, vulnerable patients most in need of social and psychiatric care, such as homeless people with addiction issues, are eclipsed from accessing supports. The high proportion of patients reporting to be homeless is cause for concern and suggests the need for tailored and integrated multi-disciplinary assessments and interventions at an Emergency Department level.

